# HMGB1 binds to and disrupts the hairpin structure of RNA15 and inhibits toll-like receptor activation

**DOI:** 10.1016/j.jbc.2026.111155

**Published:** 2026-01-12

**Authors:** Cong Lin, Penghui Li, Anna G. Savitskaya, Ekaterina Lyukmanova, Sergey A. Goncharuk, Konstantin S. Mineev, Xiubo Du, Yibo Wang, Xiaohui Wang

**Affiliations:** 1 Interdisciplinary Laboratory for Frontier Chemistry, Changchun Institute of Applied Chemistry, Chinese Academy of Sciences, Changchun, Jilin, China; 2 Shenzhen Key Laboratory of Marine Biotechnology and Ecology, College of Life Sciences & Oceanography, Shenzhen University, Shenzhen, Guangdong, China; 3 Key Laboratory of Optoelectronic Devices and System of Ministry of Education and Guangdong Province, College Physics and Optoelectronic Engineering, Shenzhen University, Shenzhen, Guangdong, China; 4 Shemyakin-Ovchinnikov Institute of Bioorganic Chemistry, Russian Academy of Sciences, Moscow, Russia; 5 Biological Department, Shenzhen MSU-BIT University, Shenzhen, Guangdong, China; 6 School of Applied Chemistry and Engineering, University of Science and Technology of China, Hefei, Anhui, China

**Keywords:** high mobility group box 1, receptor activation, remodeling, RNA, Toll-like receptor 13

## Abstract

Toll-like receptor 13 (TLR13) is a critical innate immune sensor that recognizes a conserved RNA sequence, RNA15 (2054–2068, ACG GAA AGA CCC CGU), within bacterial 23S rRNA, thereby initiating a proinflammatory response. While the alarmin high mobility group box 1 (HMGB1) is known to modulate various TLR pathways, its influence on TLR13 signaling has remained unexplored. Here, we reveal that HMGB1 directly binds RNA15 with high affinity and profoundly disrupts its hairpin structure, which is essential for TLR13 recognition. Using a combination of fluorescence anisotropy, FRET assays, NMR spectroscopy, and enhanced-sampling molecular dynamics simulations, we demonstrate that HMGB1 binding remodels RNA15 into a stem-open conformation, making it thermodynamically unfavorable for receptor activation. Functionally, HMGB1 significantly inhibits RNA15-induced TLR13 activation, leading to a dose-dependent reduction in inflammatory mediators. These findings uncover a novel “ligand remodeling” mechanism, whereby HMGB1 acts as a negative regulator of TLR13 signaling by structurally altering the RNA ligand rather than directly blocking the receptor. This work provides new insights into host–pathogen interactions and suggests important implications for the design and immunogenicity of RNA-based therapeutics and vaccines.

Toll-like receptors (TLRs) represent a critical family of proteins that serve as frontline sensors in the innate immune system, detecting conserved molecular patterns from invading pathogens. Among these receptors, Toll-like receptor 13 (TLR13) plays an essential role in host defense against bacterial infections (1). Primarily localized on the endosomal membranes of macrophages and dendritic cells, TLR13 acts as a pattern recognition receptor, specifically recognizing bacterial rRNA sequences and initiating rapid inflammatory responses (2, 3). TLR13 exhibits remarkable specificity for a conserved region of bacterial 23S rRNA spanning nucleotides 2054 to 2068, referred to herein as RNA15 (5′-ACGGAAAGACCCCGU-3′) (3, 4, 5, 6, 7). Structural studies have demonstrated that RNA15 adopts a stem–loop-like conformation when bound within the TLR13 receptor complex, stabilizing the active receptor dimer and triggering downstream signaling *via* the MyD88-and Unc93b1-dependent pathways (3, 8). This signaling cascade ultimately activates the transcription factor NF-_κ_B, leading to robust production of proinflammatory cytokines crucial for pathogen elimination (9, 10). Intriguingly, recent evidence indicates that free RNA15 in solution preferentially exists as a stable hairpin structure, inherently resistant to nuclease degradation because of its intramolecular interactions (11). This inherent structural stability of RNA15 suggests that the recognition by TLR13 likely involves a conformational adaptation or fine-tuning of the pre-existing hairpin structure, rather than a complete unfolding, to achieve the specific stem–loop configuration optimal for receptor binding, highlighting the importance of RNA ligand conformation in innate immune activation (7).

Host factors significantly influence TLR signaling pathways, and among these, high mobility group box 1 (HMGB1) protein has emerged as a prominent modulator (12). HMGB1 is a highly conserved nuclear protein traditionally involved in fundamental cellular processes, such as transcription, replication, and DNA repair. However, extracellular HMGB1 functions as a potent alarmin, amplifying inflammatory responses through interactions with multiple TLRs (12). HMGB1 is well characterized for its role in facilitating TLR4 activation. It can directly bind MD2 and also form complexes with lipopolysaccharide (LPS), thereby enhancing TLR4-mediated signaling (13, 14, 15, 16). In addition, HMGB1 contributes to TLR9-mediated immune responses by forming complexes with DNA, which facilitates the recognition and sensing of nucleotides by TLR9 (17). Notably, HMGB1 has a broad ability to bind various nucleic acids, including both DNA and RNA, often without strict sequence specificity (18). Through these interactions, HMGB1 can induce conformational changes in the nucleic acids it binds. This property suggests a potential capacity for HMGB1 to interact with RNA15 and modulate TLR13 activation.

In this study, we provide the first investigation of the direct interaction between HMGB1 and the bacterial RNA ligand RNA15. Employing biochemical assays, fluorescence-based techniques, NMR spectroscopy, and enhanced-sampling molecular dynamics (MD) simulations, we characterize the high-affinity binding interaction between HMGB1 and RNA15. Crucially, we demonstrate that this interaction profoundly alters RNA15’s conformation from its native hairpin structure to a thermodynamically favored open conformation, impairing its recognition by TLR13. Using cell-based assays, we further confirm the functional consequences of this interaction, showing that HMGB1 effectively inhibits RNA15-induced TLR13 activation and subsequent inflammatory cytokine production. Our findings reveal a novel regulatory mechanism within the innate immune response, highlighting the critical modulatory role of HMGB1 in RNA ligand recognition by TLR13. This work expands our understanding of host–pathogen interactions and underscores the importance of nucleic acid conformational dynamics in immune signaling.

## Results

**Figure 1:**
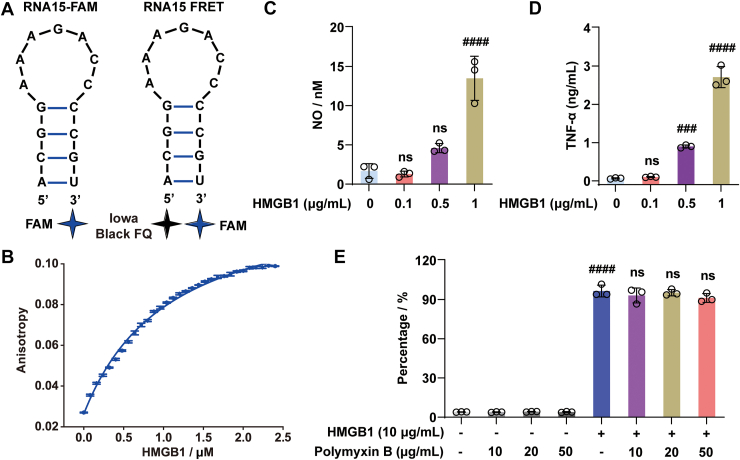
**Functional characterization of purified HMGB1.***A*, the predicted secondary structure of the RNA15-FAM and RNA15 FRET. *B*, fluorescence anisotropy titration of FAM-labeled CpG B DNA (40 nM) with increasing concentrations of HMGB1. *C* and *D*, effects of HMGB1 on the production of proinflammatory mediators NO (*C*) and TNF-α (*D*) in RAW 264.7 cells. *E*, impact of polymyxin B on HMGB1-induced NO production in BV-2 cells, confirming that the observed activity is independent of potential LPS contamination. All experiments were performed in three independent replicates (n = 3). Data were presented as the mean ± SD and analyzed by one-way ANOVA. ###*p* < 0.001, ####*p* < 0.0001 *versus* the control group; ns, not significant *versus* the control group or the HMGB1 group. HMGB1, high mobility group box 1; LPS, lipopolysaccharide; NO, nitric oxide; TNF-α, tumor necrosis factor alpha.

HMGB1 is a danger-associated molecular pattern molecule that plays a critical role in the body’s immune response. It is ubiquitously expressed in almost all eukaryotic cells, and its genetic sequence is highly conserved across species, sharing more than 98% similarity between rodents and humans (19, 20, 21). To investigate the potential interaction between HMGB1 and the TLR13 ligand RNA15 (Fig. 1*A*), HMGB1 was first recombinantly expressed and purified to ensure its biological activity. The activity of the purified protein was evaluated by measuring its binding affinity to a known ligand, CpG B DNA, using fluorescence anisotropy. As shown in Figure 1*B*, titration of fluorescently labeled CpG B DNA with our purified HMGB1 yielded a dissociation constant (*K*_*d*_) of 1.03 ± 0.07 μM, which closely matches previously reported values (∼1.1 μM) (22). This result confirms that the purified HMGB1 is functional.

The biological activity of the purified HMGB1 was next evaluated using cellular assays. Consistent with its known proinflammatory function, treatment with purified HMGB1 led to a significant, dose-dependent increase in the production of nitric oxide (NO) (Fig. 1*C*) and tumor necrosis factor-α (TNF-α) (Fig. 1*D*) in RAW264.7 cells. Similar dose-dependent increases in NO (Fig. S1*A*) and TNF-α (Fig. S1*B*) production were also observed in BV-2 cells. To exclude the possibility that this inflammatory activity was caused by LPS contamination, a common concern in recombinant protein preparations, additional assays were performed in the presence of polymyxin B, a potent LPS-neutralizing agent (23, 24). As shown in Figure 1*E*, HMGB1 continued to induce robust NO production even when polymyxin B was added at varying concentrations. The failure of polymyxin B to inhibit this response strongly indicates that the observed proinflammatory response was not because of LPS contamination but was instead attributable to the intrinsic biological activity of HMGB1. Collectively, these validation experiments confirm the successful preparation of the biologically active HMGB1, providing a reliable foundation for subsequent mechanistic studies.

**Figure 2:**
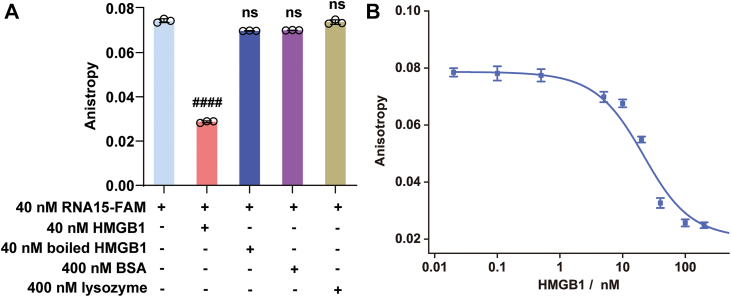
**Interaction of HMGB1 with FAM-labeled RNA15 measured by fluorescence anisotropy.***A*, binding of HMGB1 induces a specific-dependent decrease in the fluorescence anisotropy of RNA15-FAM. *B*, titration curve of RNA15-FAM with increasing concentrations of HMGB1. All experiments were performed in three independent replicates (n = 3). Data were presented as the mean ± SD and analyzed by one-way ANOVA. ####*p* < 0.0001 *versus* the control group; ns, not significant *versus* the control group. HMGB1, high mobility group box 1.

Having confirmed the functional activity of the purified HMGB1, we next investigated whether it could directly interact with RNA15, the specific ligand for TLR13. To assess this potential interaction, we measured the fluorescence anisotropy of 3′-FAM-labeled RNA15 (40 nM) in the presence of HMGB1. RNA15 is known to form a stable intramolecular hairpin structure, which restricts the rotational freedom of its terminal FAM label. As a result, the RNA15-FAM probe exhibits a relatively high initial anisotropy value (0.079; Fig. 2*A*). This value is notably higher than that of free FAM, which typically exhibits anisotropy values around 0.02. Upon addition of HMGB1, we observed a dose-dependent decrease in the fluorescence anisotropy of RNA15-FAM (Fig. 2*B*), suggesting a structural alteration of the RNA probe. The titration revealed a *K*_*d*_ of 23.7 ± 2.4 nM, indicating a strong, high-affinity interaction comparable to the affinity of RNA15 for its receptor TLR13 (15–50 nM, depending on assay conditions) (7, 11). Importantly, this effect was specific to active HMGB1, as control proteins, including heat-denatured HMGB1 (40 nM), bovine serum albumin (BSA, 400 nM), and lysozyme (400 nM), did not alter the anisotropy signal (Fig. 2*A*).

Typically, when a small fluorescently labeled ligand binds to a larger protein, the resulting increase in molecular weight slows molecular tumbling, leading to an increase in anisotropy. This expected behavior was observed with FAM-labeled CpG B DNA upon HMGB1 binding, which showed an increase in anisotropy (Fig. 1*A*). Interestingly, in contrast, we observed a decrease in anisotropy upon HMGB1 binding to RNA15-FAM. This counterintuitive result suggests that HMGB1 binding does not simply increase the mass of the complex but rather disrupts the RNA15 hairpin structure. By destabilizing the hairpin, HMGB1 likely frees the 3′-FAM label, allowing greater rotational freedom and resulting in the observed decrease in anisotropy.

**Figure 3:**
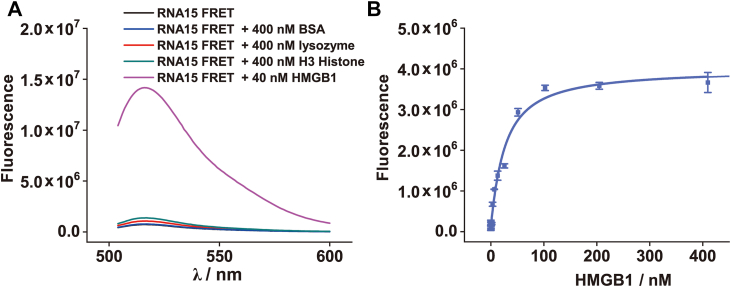
**Interaction of HMGB1 with the RNA15 FRET probe.***A*, fluorescence intensity of the RNA15 FRET probe (40 nM) in the presence of HMGB1 (40 nM), BSA (400 nM), lysozyme (400 nM) and H3 histone (400 nM). *B*, titration curve of the RNA15 FRET probe (40 nM) with increasing concentrations of HMGB1. BSA, bovine serum albumin; HMGB1, high mobility group box 1.

To directly test the hypothesis that HMGB1 remodels the structure of RNA15, we used an RNA15 FRET probe labeled with a fluorophore (FAM) at one end and a quencher (Iowa Black FQ) at the other. In its folded hairpin conformation, the close proximity of the fluorophore and quencher enables efficient FRET, resulting in low fluorescence intensity; disruption of the hairpin increases their separation, reduces FRET efficiency, and leads to increased fluorescence. Upon addition of HMGB1 (40 nM), we observed a significant fluorescence increase in the RNA15 FRET probe (40 nM; Fig. 3*A*). This effect was specific to active HMGB1, as control proteins, including BSA (400 nM), lysozyme (400 nM), and histone H3 (400 nM), did not induce comparable changes (Fig. 3*A*). The isoelectric points (pI) of BSA, lysozyme, and histone H3 are 4.7, 11.0, and 10.8, respectively; under physiological conditions, BSA is negatively charged, whereas lysozyme and histone H3 are positively charged, with histone H3 naturally binding nucleic acids. While BSA had virtually no effect, lysozyme and histone H3 caused slight fluorescence increases, though far weaker than HMGB1, with histone H3 showing a stronger effect consistent with its nucleic acid binding role. These results provide direct evidence that HMGB1 physically separates the 3′ and 5′ ends of RNA15, disrupting its hairpin and converting it to an open state. Fitting the fluorescence enhancement curve yielded a dissociation constant of 21.1 ± 3.9 nM (Fig. 3*B*), which closely matched the value from anisotropy experiments, further confirming a direct, high-affinity interaction.

**Figure 4:**
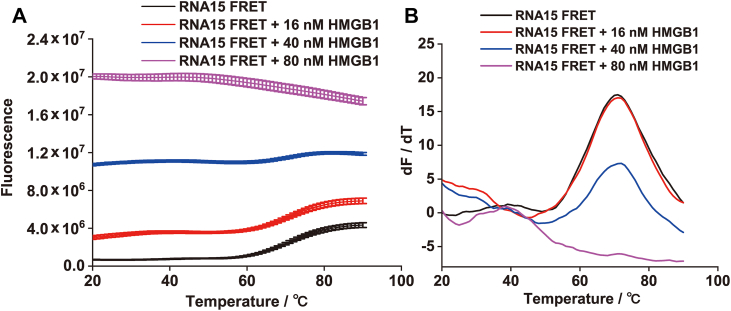
**Thermal denaturation of RNA15 FRET probe.***A*, thermal denaturation curves of the RNA15 FRET probe (40 nM) in the presence of different concentrations of HMGB1. *B*, first derivatives of the curves shown in (*A*), highlighting changes in transition profiles. HMGB1, high mobility group box 1.

Furthermore, thermal denaturation experiments were conducted to assess the impact of HMGB1 binding on RNA15 structure. As expected, heating the RNA15 FRET probe (40 nM) alone melted its hairpin, with a *T*_m_ of 71.5 ± 3.5 °C (Fig. 4*A*), leading to end separation, loss of FRET, and increased fluorescence. In the presence of increasing HMGB1 concentrations, the *T*_m_ remained unchanged, but the transition amplitude progressively decreased (Fig. 4*B*), indicating that HMGB1 disrupts the hairpin structure. At 80 nM HMGB1, no thermal transition was observed; instead, fluorescence intensity decreased with rising temperature, reflecting complete hairpin disruption and thermal quenching of the FAM fluorophore. Collectively, these results further confirm that HMGB1 effectively destabilizes and remodels the intramolecular hairpin structure of RNA15.

**Figure 5:**
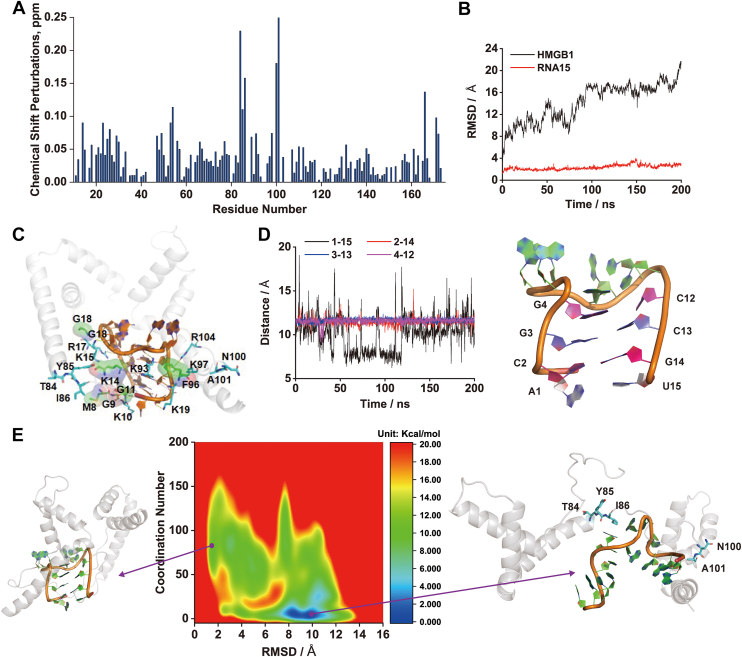
**Structural and thermodynamic analysis of the HMGB1–RNA15 interaction.***A*, amide chemical shift perturbations (CSPs) observed for HMGB1 upon RNA15 titration, highlighting the binding interface. *B*, RMSDs of the HMGB1 backbone (*black*) and RNA15 heavy atoms (*red*) during canonical MD simulations. *C*, representative binding mode of RNA15 with HMGB1 observed during MD simulations. *D*, interstrand distances between base pairs in the RNA15 stem during simulations, indicating that the hairpin structure remains intact on this timescale. *E*, free energy landscape of RNA15 conformational changes in the HMGB1-bound state derived from metadynamics, plotted as a function of RMSD from the hairpin state and the stem coordination number. Representative structures of the hairpin and stem-open states are shown. HMGB1, high mobility group box 1; MD, molecular dynamics.

To elucidate the mechanism of this interaction at an atomic level, we combined NMR spectroscopy with molecular simulations. NMR chemical shift perturbation (CSP) analysis of ^15^N-labeled HMGB1 upon titration with RNA15 revealed significant shifts for residues T84, Y85, I86, N100, and A101 (Fig. 5*A*), identifying an RNA-binding interface on the protein. These experimental data guided computational docking to generate the most plausible structural model of the HMGB1–RNA15 complex. Ten poses were generated using the 3dRPC web server, and the optimal model was selected based on the proximity of RNA15 to residues showing large CSPs. This complex was then equilibrated using 200 ns of MD simulations. As shown in Figure 5*B*, HMGB1 undergoes substantial conformational changes upon binding RNA15, whereas the RNA15 hairpin structure remains largely stable during the MD timescale. Detailed analysis revealed that HMGB1 interacts with RNA15 through hydrogen bonds involving residues G1, K10, K15, K95, K97, and R104, as well as hydrophobic interactions contributed by M8, G9, K14, G18, and F96 (Fig. 5*C*). However, the canonical MD simulations were not sufficient to capture the large-scale opening of the RNA hairpin, as interstrand distances between base pairs remained unchanged (Fig. 5*D*).

To overcome this limitation and explore the thermodynamics of hairpin unfolding, we employed well-tempered metadynamics, an enhanced sampling technique. The resulting two-dimensional potential of mean force map (Fig. 5*E*) revealed a key insight: while the hairpin is the most stable conformation for free RNA15, the presence of HMGB1 dramatically reshapes the energy landscape. In the HMGB1-bound state, a stem-open conformation characterized by a low coordination number between stem bases is thermodynamically favored by 9.86 kcal/mol over the hairpin structure. This computational result provides a robust thermodynamic explanation for the biophysical data, demonstrating that HMGB1 binding actively drives RNA15 from a closed hairpin to a stem-open state.

**Figure 6:**
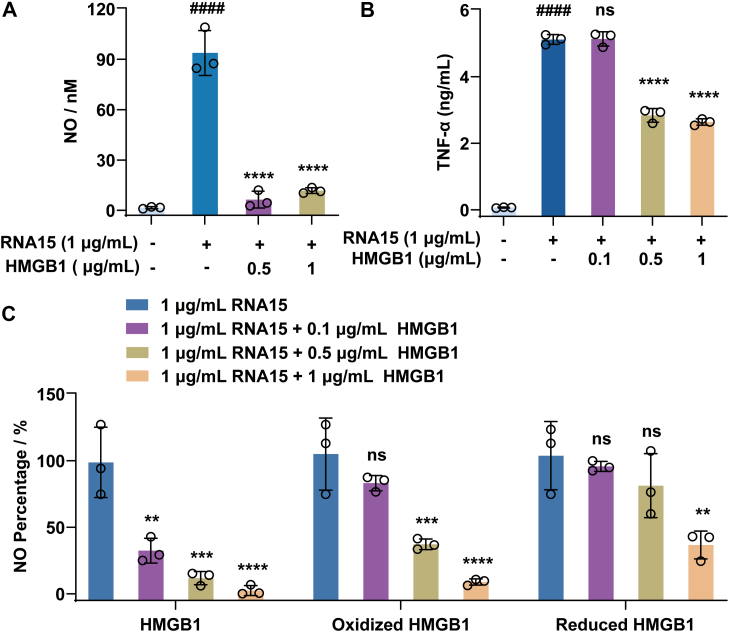
**HMGB1 inhibits RNA15-induced overexpression of inflammatory factors in RAW264.7 cells.***A*, HMGB1 suppresses RNA15-induced NO production. *B*, HMGB1 suppresses RNA15-induced TNF-α production. *C*, comparative effects of native HMGB1, H_2_O_2_-oxidized HMGB1, and DTT-reduced HMGB1 on RNA15-induced NO production. For redox modification, HMGB1 was pretreated with H_2_O_2_ (50 μM) or DTT (5 μM) for 2 h before addition to cell cultures. All experiments were performed in three independent replicates (n = 3). Data were presented as the mean ± SD and analyzed by one-way ANOVA. ####*p* < 0.0001 *versus* the control group; ∗∗*p* < 0.01, ∗∗∗*p* < 0.001, ∗∗∗∗*p* < 0.0001 *versus* the RNA15 group; ns, not significant *versus* the RNA 15 group. HMGB1, high mobility group box 1; H_2_O_2_, hydrogen peroxide; NO, nitric oxide; TNF-α, tumor necrosis factor alpha.

Given the finding that HMGB1 binds to and structurally disrupts the RNA15 hairpin, the critical conformation required for TLR13 recognition, we hypothesized that HMGB1 may inhibit RNA15-mediated TLR13 activation. As expected, stimulation with RNA15 alone robustly activated TLR13 signaling, as evidenced by significant increases in the proinflammatory mediators NO (Fig. 6*A*) and TNF-α (Fig. 6*B*). In contrast, cotreatment with RNA15 and HMGB1 markedly suppressed the induction of these factors, demonstrating a clear functional consequence of HMGB1–RNA15 binding. Notably, both oxidized HMGB1 (treated with hydrogen peroxide) and reduced HMGB1 (treated with DTT) showed a diminished ability to inhibit RNA15-induced activation compared with unmodified HMGB1 (Fig. 6*C*), highlighting the critical importance of HMGB1’s redox state in regulating its activity. By disrupting the hairpin structure necessary for TLR13 recognition, HMGB1 acts as a negative regulator of TLR13 signaling. This reveals a novel modulatory role for HMGB1 in dampening the innate immune response to bacterial RNA. Importantly, although HMGB1 is widely recognized as a proinflammatory danger-associated molecular pattern molecule that enhances TLR4 (13, 14), TLR2/6 (25), and TLR9 signaling (17), our findings uncover its unexpected inhibitory effect on TLR13 activation, adding further complexity to its immunomodulatory profile.

## Discussion

**Figure 7:**
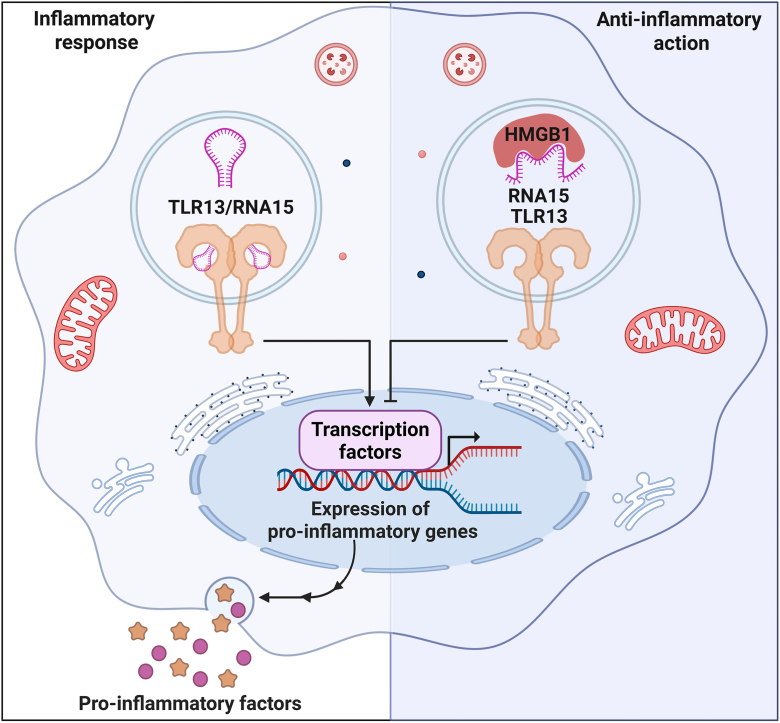
**HMGB1 inhibits TLR13 signaling by remodeling its RNA ligand.** HMGB1 binds with high affinity to RNA15, the bacterial ligand for TLR13. This interaction disrupts the essential hairpin structure of RNA15 required for receptor activation. By stabilizing a “stem-open” conformation, HMGB1 prevents RNA15 from activating TLR13, thereby suppressing downstream inflammatory responses in macrophages. HMGB1, high mobility group box 1; TLR13, Toll-like receptor 13.

This study elucidates a novel regulatory mechanism within the innate immune system, demonstrating that HMGB1 acts as a negative regulator of TLR13 signaling (Fig. 7). Through a combination of biochemical, biophysical, and computational approaches, we show that HMGB1 binds to RNA15, the bacterial RNA ligand for TLR13, with high affinity and specificity. Crucially, this interaction actively remodels RNA15, disrupting its essential hairpin structure required for TLR13 recognition. While TLR13 exhibits high specificity for the conserved RNA15 sequence from bacterial 23S rRNA, its functional activation depends critically on the secondary structure. This highlights that the mere presence of the specific sequence is insufficient; the proper conformation is the functional determinant for receptor engagement. Consequently, HMGB1’s profound inhibition of TLR13 signaling can be explained by the direct consequence of its “ligand remodeling” action. Driven by a thermodynamic preference for a stem-open conformation in the presence of HMGB1, this structural alteration diminishes RNA15’s ability to activate TLR13. By actively and thermodynamically favoring an open state, HMGB1 effectively removes the required structural “key” for TLR13 binding, thereby rendering the specific RNA15 sequence functionally inert. This results in the significant inhibition of downstream inflammatory responses in macrophages.

Our findings highlight a compelling example of “ligand remodeling” as a regulatory strategy. HMGB1 modifies the ligand’s structure, rendering it functionally inactive for receptor engagement. Initial evidence from fluorescence anisotropy and FRET assays revealed this structural disruption, which was further substantiated by molecular simulations. Notably, well-tempered metadynamics mapping of the free energy landscape provided atomic-level insights, demonstrating that HMGB1 binding makes the hairpin conformation of RNA15 thermodynamically unfavorable. This highlights the principle that the biological activity of nucleic acids is determined not only by their sequence but also by their dynamic conformational states, which can be precisely modulated by host proteins.

Our findings support an HMGB1-mediated “ligand remodeling” model in which HMGB1 disrupts the secondary structure of RNA15 that is required for TLR13 activation. However, the current assays do not directly demonstrate that an HMGB1-remodeled RNA15 species is intrinsically incapable of engaging TLR13, and alternative mechanisms remain plausible. To explore whether HMGB1 could also act as a direct competitor at the receptor, we performed protein–protein docking (Fig. S2*A*), followed by MD simulations. Among the top-ranked docking poses, we identified a stable configuration in which HMGB1 engages TLR13 at the dimerization interface, with partial overlap of the predicted footprint with the RNA15 interaction region (Fig. S2*B*). A 200-ns MD simulation supports the stability of this complex (Fig. S2*C*), consistent with a model in which HMGB1 may inhibit TLR13 signaling by sterically hindering RNA15 binding and/or preventing productive receptor dimerization. Taken together, our results support a framework in which HMGB1 can modulate TLR13 activation through multiple, potentially concurrent mechanisms, including ligand remodeling and direct competitive interference.

These findings also add a critical layer of complexity to our understanding of HMGB1 and TLR signaling. While HMGB1 is traditionally characterized as a proinflammatory alarmin (12) that enhances TLR2 (25), TLR4 (13, 14), and TLR9 (17) signaling, our work reveals its context-dependent, inhibitory role in the TLR13 pathway. This suggests that HMGB1 functions as a molecular rheostat, capable of amplifying certain immune responses, whereas dampening others. Such dual functionality may serve as a vital homeostatic mechanism to fine-tune responses to bacterial infections, preventing excessive inflammation and minimizing collateral tissue damage that could result from unchecked TLR13 activation. The identification of this intrinsic “brake” on a nucleic acid–sensing pathway highlights the sophisticated checks and balances that govern innate immunity.

Furthermore, our study has significant implications for the field of RNA therapeutics and vaccines. The efficacy and safety of RNA-based medicines depend heavily on their structural integrity and interactions with host factors. Our discovery that a ubiquitous host protein like HMGB1 can bind to and remodel short RNA molecules suggests that therapeutic or vaccine RNAs may undergo structural alterations *in vivo*, potentially affecting their stability, translational efficiency, or immunostimulatory properties. Future RNA therapeutic design may need to account for such host protein interactions, possibly through sequence engineering or chemical modifications that stabilize desired conformations and prevent off-target effects that could compromise efficacy or elicit unintended immune responses.

In summary, by demonstrating that HMGB1 inhibits TLR13 activation through remodeling of its RNA ligand, this study not only uncovers a novel dimension of innate immune regulation but also provides a critical framework for the design of next-generation RNA therapeutics. It serves as an important reminder of the dynamic interplay between host proteins and nucleic acids, with far-reaching implications for both basic immunology and translational medicine.

## Experimental procedures

### Materials

HMGB1 was prepared according to a previously reported method (26). FAM-CpG B DNA, RNA15 (ACG GAA AGA CCC CGU), RNA15-FAM (ACG GAA AGA CCC CGU-FAM), and RNA15 FRET probe (Iowa Black FQ-ACG GAA AGA CCC CGU-FAM) were synthesized and purified by Integrated DNA Technologies. BSA, lysozyme, and H3 histone were obtained from Sigma–Aldrich.

### Fluorescence measurements

Fluorescence measurements for all experiments were conducted on a Cary Eclipse spectrofluorometer. FAM-CpG B DNA, RNA15-FAM, and the RNA15 FRET probe were separately dissolved in PBS (10 mM Na_2_HPO_4_, 1.8 mM KH_2_PO_4_, 2.7 mM KCl, and 137 mM NaCl; pH = 7.4). The excitation wavelength was 494 nm. Their fluorescence intensities or fluorescence anisotropy signals at 520 nm were measured upon titrations with different concentrations of HMGB1 or other proteins. Thermal melting experiments were carried out with a heating rate of 1 °C per minute.

### NMR spectroscopy

Truncated construct of HMGB1 (HMGB1dC, residues 1–166 of human HMGB1) was cloned into a pET21d (+) plasmid, which carried a 6× His-tag sequence and tobacco etch virus-protease cleavage site. The plasmid was expressed in BL21(DE3) *Escherichia coli* strain in M9 minimal salt medium containing 4 mg/L ^15^NH_4_Cl and 50 μg/ml kanamycin. Expression was induced by 0.5 mM IPTG, and cells were cultured for 4 h at 37 °C and 250 rpm. The cell pellets were resuspended in the lysis buffer (20 mM Tris–HCl at pH 8, 0.15 M NaCl, 10 mM imidazole, and 2 mM β-mercaptoethanol) containing 0.2% Triton X-100, 0.2 mM PMSF, and 20 U/ml benzonase, and lysed by sonication. The supernatant after lysis was purified by nickel–nitrilotriacetic acid affinity chromatography (elution by 20 mM Tris–HCl at pH 8, 0.15 M NaCl, 2 mM β-mercaptoethanol, and 250 mM imidazole). The hexahistidine tag was cleaved by tobacco etch virus-protease overnight in the restriction buffer (20 mM Tris at pH 8.0, 200 mM NaCl, and 1 mM DTT). HMGB1dC was purified by the reverse affinity chromatography on nickel–nitrilotriacetic acid resin column. Finally, the sample was applied to a Superdex 75 Increase 10/300 GL column (Cytiva) in the SEC buffer (20 mM Tris–HCl at pH 8.0, 0.2 M NaCl, and 1 mM DTT), only the HMGB1dC monomer (nucleic acid free) fraction was used for further NMR experiments.

For the NMR experiments, the protein was transferred to the NMR buffer: 20 mM NaPi (pH 7.0), 100 mM NaCl, 3 mM DTT, and 0.02% NaN_3_. The concentration of protein in the final sample was equal to 68 μM, according to UV–visible. RNA 2054 to 2068 was dissolved in the same buffer at a concentration of 3.3 mM; the concentration of RNA was controlled by quantitative NMR.

To test the HMGB1dC–RNA interaction, the aliquots of 3.3 mM RNA15 solution were added to the ^15^N-labeled HMGB1dC sample to get the following RNA–protein ratios: 0.6, 1.2, 1.9, 2.5, 3.8, 5.0, and 10.1. At each point, we recorded the ^1^H,^15^N-heteronuclear single quantum coherence spectrum of HMGB1dC at 296 K (23 °C), using the 800 MHz Bruker Avance III NMR spectrometer, equipped with the triple-resonance cryogenic probe. The chemical shift assignment of HMGB1dC was taken from the Biological Magnetic Resonance Databank ID 11147, which contains the data for the same protein construct under the same ambient conditions. Then, chemical shifts were tracked in the course of the titration procedure, and values, obtained at RNA15–HMGB1dC 1.9:1 ratio, were compared to the chemical shifts in the apo state. We used the generalized chemical shift changes of amide groups as CSPs:

$$\text{CSP}=\sqrt{\delta H^{2}+\delta N^{2}/36}$$

where δH and δN are the chemical shift changes of proton and nitrogen of an amide group, and the value 36 was used to account for the difference in ^1^H and ^15^N chemical shift dispersion.

### MD simulations

The initial coordinate for HMGB1 was obtained from the Protein Data Bank (2YRQ). The structure of RNA15 hairpin was taken from our previous study, which was equilibrated in solvent for 100 ns (11). To generate a model of the HMGB1–RNA15 complex, the protein and RNA structures were submitted to the 3dRPC web server for docking prediction. From the resulting docked poses, a final model was selected based on its structural agreement with experimental NMR CSP data, ensuring that the predicted protein–RNA interface was consistent with observed interactions.

The selected HMGB1–RNA15 complex structure was further relaxed *via* MD simulations. The complex was solvated in a cubic box with dimensions of approximately 74 × 74 × 74 Å^3^ with the TIP3P water model using the CHARMM-GUI web server (27). The system was neutralized and brought to a physiological ionic strength of 150 mM with KCl ions. The CHARMM36m force field was applied for the protein, and the CHARMM36 force field was used for the nucleic acid (28, 29). The MD simulations for equilibrium were performed using NAMD 2.12 (30) in an isotropic (NPT) ensemble. The temperature was maintained at 310.15 K using the Nosé–Hoover thermostat (31), and the pressure was kept at 1 atm using the Langevin piston method (32). Periodic boundary conditions were applied throughout all simulations. Long-range electrostatic interactions were treated by the particle-mesh Ewald algorithm, with nonbonded interactions being switched off between 10 and 12 Å (33).

To investigate the conformational transition of RNA15 between its hairpin and stem-open states in the presence of HMGB1, we computed the free energy landscape using well-tempered metadynamics simulations (34, 35). This enhanced sampling technique accelerates the exploration of conformational space by applying a history-dependent biasing potential along specific collective variables, allowing the system to overcome high-energy barriers.

Two collective variables were chosen as reaction coordinates to map the RNA’s structural transformation and reconstruct the potential of mean force. These include the RMSD calculated for the RNA backbone atoms relative to the initial, equilibrated hairpin conformation, which tracks the overall structural deviation from the starting state, and the coordination number defined between the first four and the last four bases of the RNA15 sequence, which specifically monitors the formation and dissociation of the hairpin stem. Specifically, the coordination numbers reflect the strength of interaction between the two atom groups, defined as:

$${CN}_{\text{Total}}=\sum_{i\in A}\sum_{j\in B}{CN}_{ij}$$

where *CN*_*ij*_ is the coordination number between atom *i* in group A (bases 1–4) and atom *j* in group B (bases 12–15), given by the switching function:

$${CN}_{ij}=\frac{1-{\left(\frac{r_{ij}-d_{0}}{r_{0}}\right)}^{6}}{1-{\left(\frac{r_{ij}-d_{0}}{r_{0}}\right)}^{12}}$$

Here, *r*_*ij*_ is the distance between atoms *i* and *j*, and the cutoff distance *r*_*0*_ was set to 3.0 Å.

During the metadynamics simulation, Gaussian hills with an initial height of 1.0 kJ/mol were added every 0.2 ps. The widths of the Gaussians were set to 0.5 Å for the RMSD and 10 for the dimensionless coordination number. A bias factor of 15 was used for the well-tempered protocol (34, 35). A total simulation time of 92 ns was performed to ensure the convergence of the free energy landscape.

The metadynamics simulations were performed using the graphics processing unit–accelerated version of Amber 22 (36) in conjunction with the PLUMED 2.8.0 plugin (37, 38, 39). Simulations were conducted in the NPT ensemble, maintaining the temperature at 300 K with a Langevin thermostat and the pressure at 1 atm. A 2 fs integration timestep was enabled by constraining all bonds involving hydrogen atoms with the SHAKE algorithm (40, 41), and the Velocity-Verlet algorithm was used to integrate the equations of motion (42). Trajectory visualization and figure generation were performed using the open-source software PyMOL 2.5 (Schrödinger LLC) (43).

### Cellular assays

RAW 264.7 cells and BV-2 cells were obtained from the China Center for Type Culture Collection. Cell line authentication for both BV-2 and RAW 264.7 was performed using the short tandem repeat method. RAW 264.7 cells were cultured in RPMI1640 medium supplemented with 10% fetal bovine serum, 50 U/ml penicillin, and 50 μg/ml streptomycin. When the cells reached approximately 80% confluence, they were detached from the flask using trypsin digestion. BV-2 cells were maintained in Dulbecco's modified Eagle's medium supplemented with 10% fetal bovine serum, 50 U/ml penicillin, and 50 μg/ml streptomycin and were detached using a cell lifter upon reaching ∼80% confluence. Before experiments, all cell cultures were confirmed to be free of mycoplasma contamination.

Cells were then seeded into 96-well plates at a density of 4 × 10^4^ cells per well for BV-2 cells and 8 × 10^4^ cells per well for RAW 264.7 cells. After overnight incubation, the complete medium was replaced with serum-free medium. Subsequently, the cells were treated with RNA15, different concentrations of HMGB1, or a combination of RNA15 (1 μg/ml, ∼200 nM) and HMGB1 (at a maximum concentration of 1 μg/ml, ∼40 nM). Prior to treatment, RNA15 and HMGB1 were premixed and incubated at room temperature for 30 min. Following this, cells were treated with varying concentrations of RNA15, HMGB1, or a combination of RNA15 and HMGB1. Following 24 h of treatment, the levels of TNF-α in the supernatant were measured using an ELISA kit. NO levels were determined using the 2,3-diaminonaphthalene method, as previously described (44).

### Statistical analysis

Data were presented as mean ± SD. Statistical analysis was performed using one-way ANOVA in GraphPad Prism 8.0 (Dotmatics). Concentration–response curves were determined by logistic regression using Origin 8 (OriginLab Corporation). Statistical significance (*p* < 0.05) was indicated on the bars in each figure.

## Data availability

The data that support the findings of this study are included in the article or are available from the corresponding authors upon request.

## Supporting information

This article contains supporting information.

## Conflict of interest

The authors declare that they have no conflicts of interest with the contents of this article.

## References

[1] Takeuchi O., Akira S. (2010). Pattern recognition receptors and inflammation. Cell.

[2] Hidmark A., von Saint Paul A., Dalpke A.H. (2012). Cutting edge: TLR13 is a receptor for bacterial RNA. J. Immunol..

[3] Li X.D., Chen Z.J.J. (2012). Sequence specific detection of bacterial 23S ribosomal RNA by TLR13. Elife.

[4] Oldenburg M., Kruger A., Ferstl R., Kaufmann A., Nees G., Sigmund A. (2012). TLR13 recognizes bacterial 23S rRNA devoid of erythromycin resistance-forming modification. Science.

[5] Lind N.A., Rael V.E., Pestal K., Liu B., Barton G.M. (2022). Regulation of the nucleic acid-sensing toll-like receptors. Nat. Rev. Immunol..

[6] Shimizu T. (2017). Structural insights into ligand recognition and regulation of nucleic acid-sensing toll-like receptors. Curr. Opin. Struct. Biol..

[7] Song W., Wang J., Han Z.F., Zhang Y.F., Zhang H.Q., Wang W.G. (2015). Structural basis for specific recognition of single-stranded RNA by toll-like receptor 13. Nat. Struct. Mol. Biol..

[8] Pelka K., Bertheloot D., Reimer E., Phulphagar K., Schmidt S.V., Christ A. (2018). The chaperone UNC93B1 regulates toll-like receptor stability independently of endosomal TLR transport. Immunity.

[9] Sulthana S., Basturea G.N., Deutscher M.P. (2016). Elucidation of pathways of ribosomal RNA degradation: an essential role for RNase E. RNA.

[10] Walker A.S., Russ W.P., Ranganathan R., Schepartz A. (2020). RNA sectors and allosteric function within the ribosome. Proc. Natl. Acad. Sci. U.S.A..

[11] Wang Y.B., Li P.H., Wang H.S., Wang X.H. (2025). Recognition mechanism of RNA by TLR13: structural insights and implications for immune activation. J. Mol. Biol..

[12] Ren W.X., Zhao L., Sun Y., Wang X.C., Shi X.G. (2023). HMGB1 and toll-like receptors: potential therapeutic targets in autoimmune diseases. Mol. Med..

[13] Yang H., Wang H., Andersson U. (2020). Targeting inflammation driven by HMGB1. Front. Immunol..

[14] Yang H., Wang H.C., Ju Z.L., Ragab A.A., Lundbäck P., Long W. (2015). MD-2 is required for disulfide HMGB1-dependent TLR4 signaling. J. Exp. Med..

[15] Youn J.H., Oh Y.J., Kim E.S., Choi J.E., Shin J.S. (2008). High mobility group box 1 protein binding to lipopolysaccharide facilitates transfer of lipopolysaccharide to CD14 and enhances lipopolysaccharide-mediated TNF-α production in human monocytes. J. Immunol..

[16] Wähämaa H., Schierbeck H., Hreggvidsdottir H.S., Palmblad K., Aveberger A.C., Andersson U. (2011). High mobility group box protein 1 in complex with lipopolysaccharide or IL-1 promotes an increased inflammatory phenotype in synovial fibroblasts. Arthritis Res. Ther..

[17] Ivanov S., Dragoi A.M., Wang X., Dallacosta C., Louten J., Musco G. (2007). A novel role for HMGB1 in TLR9-mediated inflammatory responses to CpG-DNA. Blood.

[18] Müller S., Scaffidi P., Degryse B., Bonaldi T., Ronfani L., Agresti A. (2001). The double life of HMGB1 chromatin protein:: architectural factor and extracellular signal. EMBO J..

[19] Bustin M., Lehn D.A., Landsman D. (1990). Structural features of the HMG chromosomal proteins and their genes. BBA-Gene Struc. Exp..

[20] Štros M., Dixon G.H. (1993). A retropseudogene for non-histone chromosomal protein HMG-1. BBA-Gene Struc. Exp..

[21] Bustin M., Reeves R. (1996). High-mobility-group chromosomal proteins: architectural components that facilitate chromatin function. Prog. Nucleic Acid Res. Mol. Biol..

[22] Yanai H., Chiba S., Ban T., Nakaima Y., Onoe T., Honda K. (2011). Suppression of immune responses by nonimmunogenic oligodeoxynucleotides with high affinity for high-mobility group box proteins (HMGBs). Proc. Natl. Acad. Sci. U.S.A..

[23] Cardoso L.S., Araujo M.I., Goes A.M., Pacifico L.G., Oliveira R.R., Oliveira S.C. (2007). Polymyxin B as inhibitor of LPS contamination of Schistosoma mansoni recombinant proteins in human cytokine analysis. Microb. Cell. Fact.

[24] Trimble M.J., Mlynarcik P., Kolar M., Hancock R.E. (2016). Polymyxin: alternative mechanisms of action and resistance. Cold Spring Harb. Perspect. Med..

[25] Herzog C., Lorenz A., Gillmann H.J., Chowdhury A., Larmann J., Harendza T. (2014). Thrombomodulin's lectin-like domain reduces myocardial damage by interfering with HMGB1-mediated TLR2 signalling. Cardiovasc. Res..

[26] Li J., Wang H., Mason J.M., Levine J., Yu M., Ulloa L. (2004). Recombinant HMGB1 with cytokine-stimulating activity. J. Immunol. Methods.

[27] Jo S., Kim T., Iyer V.G., Im W. (2008). CHARMM-GUI: a web-based graphical user interface for CHARMM. J. Comput. Chem..

[28] Best R.B., Zhu X., Shim J., Lopes P.E., Mittal J., Feig M. (2012). Optimization of the additive CHARMM all-atom protein force field targeting improved sampling of the backbone phi, psi and side-chain chi(1) and chi(2) dihedral angles. J. Chem. Theor. Comput..

[29] Klauda J.B., Venable R.M., Freites J.A., O'Connor J.W., Tobias D.J., Mondragon-Ramirez C. (2010). Update of the CHARMM all-atom additive force field for lipids: validation on six lipid types. J. Phys. Chem. B.

[30] Phillips J.C., Braun R., Wang W., Gumbart J., Tajkhorshid E., Villa E. (2005). Scalable molecular dynamics with NAMD. J. Comput. Chem..

[31] Martyna G.J., Tobias D.J., Klein M.L. (1994). Constant-pressure molecular-dynamics algorithms. J. Chem. Phys..

[32] Feller S.E., Zhang Y.H., Pastor R.W., Brooks B.R. (1995). Constant-pressure molecular-dynamics simulation - the langevin piston method. J. Chem. Phys..

[33] Essmann U., Perera L., Berkowitz M.L., Darden T., Lee H., Pedersen L.G. (1995). A smooth particle mesh ewald method. J. Chem. Phys..

[34] Barducci A., Bussi G., Parrinello M. (2008). Well-tempered metadynamics: a smoothly converging and tunable free-energy method. Phys. Rev. Lett..

[35] Sutto L., Marsili S., Gervasio F.L. (2012). New advances in metadynamics. Wiley Interdiscip. Rev.-Comput. Mol. Sci..

[36] Case D.A., Aktulga H.M., Belfon K., Ben-Shalom I.Y., Berryman J.T., Brozell S.R. (2023).

[37] consortium, P (2019). Promoting transparency and reproducibility in enhanced molecular simulations. Nat. Methods.

[38] Tribello G.A., Bonomi M., Branduardi D., Camilloni C., Bussi G. (2014). Plumed 2: new feathers for an old bird. Comput. Phys. Commun..

[39] Bonomi M., Branduardi D., Bussi G., Camilloni C., Provasi D., Raiteri P. (2009). PLUMED: a portable plugin for free-energy calculations with molecular dynamics. Comput. Phys. Commun..

[40] Sindhikara D.J., Kim S., Voter A.F., Roitberg A.E. (2009). Bad seeds sprout perilous dynamics: stochastic thermostat induced trajectory synchronization in biomolecules. J. Chem. Theor. Comput..

[41] Ryckaert J.P., Ciccotti G., Berendsen H.J.C. (1977). Numerical-integration of Cartesian equations of motion of a system with constraints - molecular-dynamics of N-alkanes. J. Comput. Phys..

[42] Grubmüller H., Heller H., Windemuth A., Schulten K. (1991). Generalized verlet algorithm for efficient molecular dynamics simulations with long-range interactions. Mol. Simul..

[43] (2025). The PyMOL Molecular Graphics System; Version 2.5.

[44] Li H., Peng Y., Lin C., Zhang X., Zhang T., Wang Y. (2021). Nicotine and its metabolite cotinine target MD2 and inhibit TLR4 signaling. Innovation.

