# Supplementary material for: HMGB1 binds to and disrupts the hairpin structure of RNA15 and inhibits toll-like receptor activation
Source: J Biol Chem. 2026 Jan 12;302(3):111155. doi: 10.1016/j.jbc.2026.111155 (PMC12887398; doi:10.1016/j.jbc.2026.111155)
Supplement: Supporting information [file mmc1.docx]

Supporting Information for

**HMGB1 Binds to and Disrupts the Hairpin Structure of RNA15 and Inhibits Toll-like Receptor Activation**

Cong Lin^1#^, Penghui Li^2,3#^, Anna G. Savitskaya^4^, Ekaterina Lyukmanova^4,5^, Sergey A. Goncharuk^4^, Konstantin S Mineev^4,†^, Xiubo Du^2^, Yibo Wang^1*^, Xiaohui Wang^1,6*^

**This document includes:**

Figure S1..…..……......................…..……......…..…….................................Page S2

Figure S2..…..……......................…..……......…..…….................................Page S3


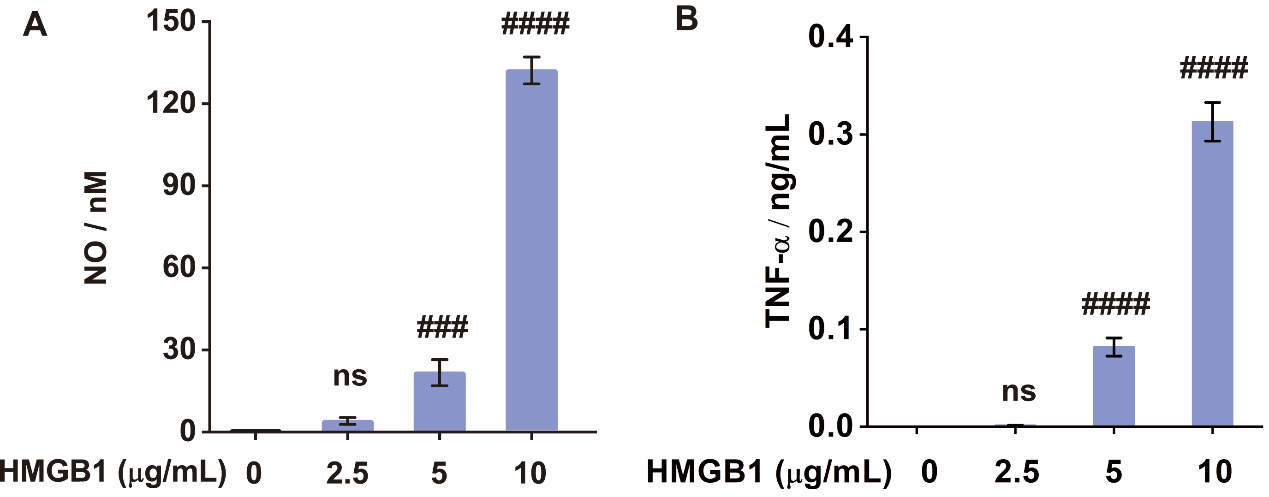


**Figure S1**. Effects of HMGB1 on the production of proinflammatory mediators nitric oxide (NO) (A) and tumor necrosis factor-α (TNF-α) (B) in BV-2 cells. All experiments were performed at least three times independently. Data were presented as the mean ± SD and analyzed by one-way ANOVA. ### *p* < 0.001, #### *p* < 0.0001 versus the control group; ns, not significant versus the control group.


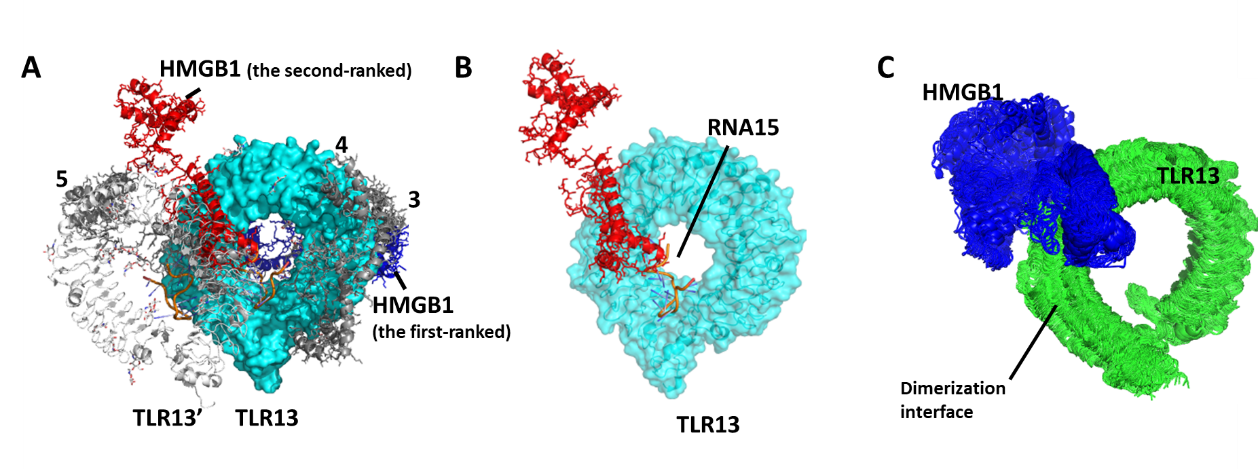


**Figure S2. Predicted binding modes of HMGB1 on TLR13 and their stability in MD simulations**. (A) The five top-scoring docking poses for HMGB1 mapped onto the TLR13 monomer; the TLR13 dimerization interface is indicated by displaying the second TLR13 protomer from the TLR13 dimer structure (PDB ID: 4Z0C). (B) Close-up of the representative binding mode (second-ranked pose) after removing the front TLR13 protomer for clarity. (C) Binding-mode stability assessed by superimposition of 100 frames extracted at 2-ns intervals from a 200-ns MD trajectory of the HMGB1–TLR13 complex.
